# Development of Al/Mg Bimetal Processed by Ultrasonic Vibration-Assisted Compound Casting: Effects of Ultrasonic Vibration Treatment Duration Time

**DOI:** 10.3390/ma16145009

**Published:** 2023-07-15

**Authors:** Qingqing Li, Feng Guan, Yuancai Xu, Zheng Zhang, Zitian Fan, Wenming Jiang

**Affiliations:** State Key Laboratory of Materials Processing and Die & Mould Technology, School of Materials Science and Engineering, Huazhong University of Science and Technology, Wuhan 430074, China

**Keywords:** ultrasonic vibration treatment, compound casting, Al/Mg bimetal, interfacial microstructure, mechanical properties

## Abstract

In this work, ultrasonic vibration treatment (UVT) was introduced to improve the interfacial microstructure and bonding strength of A356/AZ91D bimetal processed via lost foam compound casting (LFCC). The interfacial microstructure and mechanical properties of the Al/Mg bimetal processed via LFCC with different UVT durations were investigated. Results revealed the UVT did not change the composition of phases at the interface. The Al/Mg bimetallic interface consisted of an intermetallic compound area (β-Al_3_Mg_2_ + γ-Al_12_Mg_17_ + Mg_2_Si) and eutectic area (δ-Mg + γ-Al_12_Mg_17_ + Mg_2_Si). When the duration of the UVT was increased, the gathered Mg_2_Si particles at the intermetallic compound area were refined to sizes of no more than 5 μm and became more homogeneously dispersed in the intermetallic compound area and diffused in the eutectic area, which could be attributed to the removal of oxide film and the acoustic cavitation and streaming flow effects induced by the UVT. The microhardness of the Al/Mg bimetallic interface was not obviously changed by the increase in UVT duration. The shear strength of the Al/Mg bimetal was increased with UVT and reached maximum with a UVT duration of 5 s, with a value of 56.7 MPa, which was increased by 70.3%, compared with Al/Mg bimetal without UVT. This could be attributed to the removal of the oxide film at the Al/Mg bimetallic interface, which improved the metallurgical bonding of the Al/Mg interface. Additionally, the refined and homogeneously dispersed Mg_2_Si particles played an important role in suppressing the propagation of cracks and enhancing the shear strength of the Al/Mg bimetal.

## 1. Introduction

Magnesium, aluminum, and their alloys possess attractive advantages, such as low weight (*ρ*_Mg_~1.736 g/cm^3^, *ρ*_Al_~2.698 g/cm^3^), high specific strength, and excellent casting performance, which have attracted more and more attention and the metals are widely used in the automotive, marine, aerospace, and electronic fields [1,2,3,4]. Al/Mg bimetallic composites are expected to utilize the positive advantages of Mg alloys and Al alloys, such as the damping capacity of Mg alloys and corrosion resistance of Al alloys [3,5,6,7,8], which would expand their application prospects.

Different processes, such as rolling [9,10], friction stir-welding [11,12], extrusion [13,14], and compound casting [15,16,17], could be used to prepare Al/Mg bimetal. Among them, it is noteworthy that compound casting is suitable for fabricating Al/Mg bimetal objects with complex shapes and large sizes. Lost foam compound casting (LFCC) is a precise, simple, and cost-effective casting technology. During LFCC, an expanded polystyrene (EPS) foam model is prepared based on the desired size and shape of the component, then coated with refractory slurry and placed in a sandbox with loose and dry sand which is later compacted via vibration. During the pouring process, the EPS model is degraded and filled with liquid metal. After the liquid metal has solidified, the precise desired components are obtained [15,18]. Compared with the liquid–liquid LFCC process, a solid insert, as a part of the Al/Mg bimetallic component, is used in the solid–liquid LFCC process. Using a solid insert to prepare Al/Mg bimetallic components makes the reaction layer of Al alloy and Mg alloy effectively limited to the surface of the solid insert. In particular, the solid insert can be pre-mounted with the EPS model and has no need for additional fixation. Nevertheless, the dendrites of β-Al_3_Mg_2_ and γ-Al_12_Mg_17_ intermetallic compounds (IMCs) and oxide layers formed at the Al/Mg bimetallic interface during casting process are detrimental to the mechanical properties [8,19].

The dendrites of Al-Mg IMCs perpendicular to the Al/Mg bimetallic interface with high microhardness are coarse and brittle, which are easy to break up and can aid in crack initiation and propagation during deformation, thereby sharply decreasing the bonding strength of the Al/Mg bimetal. Al-Mg IMCs could be reduced or eliminated by introducing an interlayer, such as Zn [15], Ni [20,21], or Ti [22], between the Al alloy and Mg alloy. However, the preparation process for fabricating the interlayer is essential to the bonding strength of Al/Mg bimetal. For example, Li et al. [21] prepared Ni interlayers on the surface of an Al alloy insert via high-velocity oxygen fuel spraying and found that although Al-Mg IMCs were reduced or eliminated, the existence of Ni interlayers could not guarantee the enhancement of the bonding strength of the Al/Mg bimetal. The Ni interlayer fabricated with a smooth surface was not easily wetted by the liquid Mg melt. If the thickness of the Ni interlayer was too thin or too thick [21], the bonding strength of the Al/Mg bimetal would sharply decrease. Meanwhile, the preparation process of the interlayer is usually complicated and costly. Systematic studies on developing effective as well as low-cost interlayers should be conducted on the basis of a good understanding of the interface formation process between Al alloy and Mg alloy.

The aluminum oxide layers at the Al/Mg bimetallic interface with high melting point are continuous, dense, thermodynamically stable, and not easily wetted by molten metals, which would inhibit direct contact and bonding between Al alloy and Mg alloys [8]. Various efforts have been made, with limited success, to rupture the oxide layers [8,23]. The removal of the oxide layers from the interface of the bimetal needs the liquid flow in the melt to be strong enough during the casting process [24]. However, due to the chilling effect of the cold solid insert, the temperature of the liquid melt near the surface of the solid insert drops sharply, leading to limited and insufficient flow velocity at the solid-liquid interface. Studies on rupturing and removing the oxide layers at the bimetal interface are worthy of great concern.

The application of ultrasonic vibration treatment (UVT) in the casting process is a valid and pollution-free solution to the problems of refining grains, reducing porosity, and improving the chemical homogeneity of metals and alloys [25,26,27,28,29]. Ultrasonic irradiation in metallic liquid could induce acoustic cavitation and acoustic streaming flow, which has been directly observed via in situ high-speed synchrotron X-ray radiography [26,29,30]. Acoustic cavitation refers to the formation, growth and collapse of small cavities/bubbles in the liquid, as a result of cycles of tensile and compressive stresses induced by ultrasonic waves [26,29,31]. The whole life cycle of bubble nucleation, expansion and implosion could be rapidly completed in 33.3 μs. Furthermore, each implosion creates more bubble nuclei for the next ultrasound period [29]. A micro-jet of liquid rushes into the imploded bubbles at a speed of roughly 110 m/s and creates a local hot spot with a temperature of ~5500 °C, pressure of 100 MPa and heating/cooling rate of >10^10^ °C/s [32], which could fragment the growth front of columnar dendrites at the liquid–solid interface [33] as well as improve the wettability of impurity particles and activate them to become effective nuclei [34]. An acoustic streaming flow is a quasi-steady flow which is induced by the energy loss of acoustic wave propagation and driven by cavitation zone pulsation [26,31]. The imploded bubbles and the high-speed acoustic flow (~0.5 m/s [25,26]) could effectively break up the solidifying phases and the liquid–solid interface. Guan et al. [35] found the Al-Mg IMCs at the interface of the Al/Mg bimetal were refined and the continuous oxide layers were broken and eliminated by UVT at a relatively low input power (50 W), which enhanced bonding strength by 86.5%, compared with Al/Mg bimetal without UVT. Applying UVT in fabricating Al/Mg bimetal with high mechanical performance, especially high bonding strength, via compound casting is a potentially attractive method.

Ultrasonic vibration treatment (UVT)-assisted compound casting is usually applied during melt treatment [27] in order to refine grains and reduce segregation during solidification. However, studies related to UVT-assisted compound casting process to fabricate bimetallic composites are strongly limited [24,36,37], let alone those related to Al/Mg bimetallic composites. And the mechanisms of UVT in improving the interfacial microstructure and bonding strength of Al/Mg bimetal have not been systematically studied and clearly demonstrated [35]. In this work, UVT-assisted LFCC was conducted to fabricate A356/AZ91D bimetal. The effects and mechanisms of UVT at different durations on interfacial microstructure and mechanical properties were investigated, which will provide a novel potential approach to fabricating high-performance bimetallic components in industry.

## 2. Material and Method

### 2.1. Material Preparation

**Figure 1:**
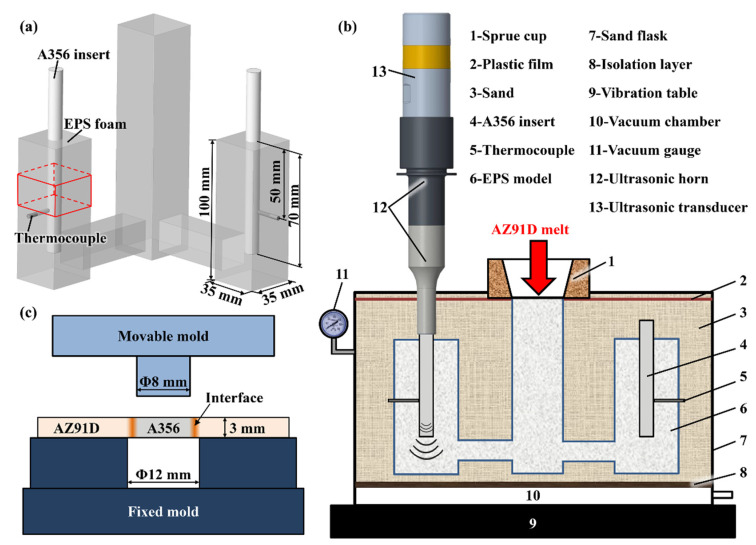
Schematic diagrams showing (**a**) the assembled EPS model (the red box marked in the image illustrates the position of the fabricated Al/Mg bimetal samples cut for microstructure observation and mechanical testing), (**b**) the UVT-assisted LFCC system, (**c**) shear strength testing.

Commercial A356 aluminum and AZ91D magnesium alloys ingots were used as the raw materials to prepare the Al/Mg bimetal. The chemical compositions of the A356 and AZ91D alloys measured by energy dispersive spectroscopy (EDS) are listed in Table 1. A rod with a diameter of 10 mm and a height of 129 mm, shaped from A356 alloy and cut using a wire electrical discharge machine, was used as the solid insert. The surface of the A356 inserts were ground with silicon carbide papers from 240 grit to 2000 grit and washed with acetone in ultrasonic cleaners for 10 min. To remove the oxide film, the A356 inserts were immersed in lye (10 g/L NaOH) and a pickling solution (50% HF + 50% HNO_3_, volume ratio) for 20–40 s, respectively. Finally, the A356 inserts were washed with anhydrous alcohol and dried, then assembled with the expanded polystyrene (EPS, 12 kg/m^3^) foam model, as shown in Figure 1a. The assembled EPS foam model consisted of a sprue (35 × 35 × 160 mm^3^), an inner sprue (22 × 45 × 22 mm^3^) and two cubes (35 × 35 × 100 mm^3^) embedded with the A356 inserts. A water-based ceramic slurry with refractory properties was brushed and coated onto the surface of the assembled EPS foam model, and then dried in an air oven. K-type thermocouples (error values ± 0.75%) with 3 mm diameter stainless steel sheaths were embedded in the side of the coated foam (Figure 1a) to measure and record the temperature variation of the bonding interface of the Al/Mg bimetal during the LFCC process. The thermocouples were connected to a computer data collection system (National Instruments, Austin, TX, USA, data acquisition frequency 75 Hz).

The UVT-assisted LFCC system is shown in Figure 1b. The ultrasonic generator (YPJ13-U040, Hangzhou Successful Ultrasound Equipment Co., Ltd., Hangzhou, China) provides a maximum output power of 2000 W and a constant frequency of 20 ± 0.5 kHz. The two A356 inserts were embedded into the EPS foam at a depth of 70 mm (Figure 1a). Specially, the top of one A356 insert was gripped and bolted by the ultrasonic horn, which could conduct UVT and as the ultrasonic radiator during casting, as shown in Figure 1b. For comparison, another A356 insert was not gripped by the ultrasonic horn. The length (*L*) of the A356 inserted into the ultrasonic horn was 129 mm, as calculated by Equation (1):

(1) $$L=\frac{1}{2f}\cdot \sqrt{\frac{E}{\rho }}$$

where *f* is the frequency of ultrasonic vibration (20 kHz), *E* is the Young’s modulus of the A356 alloy (72.4 GPa) and *ρ* is the density of A356 alloy (2710 kg/m^3^).

Subsequently, the assembled EPS foam model was placed in a sandbox and filled with loose and dry sand. The loose and dry sand was compacted using a vibration table (XF/ZDT-50VT, Xiangfeng Instrument, Changshu, China) with vibration frequency of 25 Hz and covered with a plastic film, prepared for the pouring process. When the AZ91D alloy ingot was heated and smelted in an electric resistance furnace (SG2-7.8-10, Wuhan Yahua electric furnace Co., Ltd., Wuhan, China) at a temperature of 750 °C under a shielding gas of CO_2_ + SF_6_ with a volume ratio of 95:1, the surface scum of the AZ91D melt was skimmed by a slag spoon. The pouring process was immediately carried out when the melt had cooled to 730 °C. During the pouring process, the sandbox was under 0.03 MPa vacuum pressure. It is noteworthy that the theoretical liquidus temperatures of A356 alloy (615 °C) and AZ91D alloy (598 °C) are relatively close, which leads to great challenges in performing UVT at elevated temperatures (>598 °C) due to the severe melting of the A356 insert. To avoid severe melting of the A356 insert, the temperature at which UVT was performed were set at 570 °C. When the temperature obtained via the thermocouples dropped down to 570 °C (about 90 s after the pouring process), the ultrasonic device was activated with an output power of 75 W (before pouring the AZ91D melt), a frequency of 20 kHz and a duration of 1 s. Finally, the Al/Mg bimetallic samples (35 × 35 × 100 mm^3^) as the solid A356 insert surrounded by the solidified AZ91D melt with and without ultrasonic vibration treatment, respectively, were fabricated. By changing the UVT duration while the other experimental conditions remained consistent, the Al/Mg bimetal with UVT duration of 5 s and 9 s were fabricated, respectively.

There were four kinds of samples, the as-cast Al/Mg bimetal without UVT (UVT-0) and the Al/Mg bimetals with UVT for a duration of 1 s (UVT-1), 5 s (UVT-5) and 9 s (UVT-9), respectively. The microstructure and mechanical properties of the four kinds of samples were further studied.

### 2.2. Microstructure Characterization

The red boxes near the thermocouple marked in Figure 1a illustrate the position of the fabricated Al/Mg bimetallic samples for the observation of interface microstructures. The Al/Mg bimetallic samples were cut into blocks using a wire electrical discharge machine along the directions that parallel and perpendicular to the A356 insert axis. The specimens were ground up with silicon carbide paper to 2000 grit, polished with Al_2_O_3_ suspension from 1.5 μm to 0.5 μm and etched with a 4% nital solution. The macroscopic morphology of the Al/Mg bimetallic interfaces was observed via optical microscope (OM, Keyence, Osaka, Japan). The detailed microstructures Al/Mg bimetallic interfaces, such as elemental distribution, grain/phase morphology, distribution and size, were studied using a scanning electron microscope (SEM, FEI-200F, Eindhoven, The Netherlands) equipped with an energy dispersive spectroscopy (EDS). Additionally, the fracture and crack morphology after shear strength testing was observed via SEM.

### 2.3. Mechanical Properties

Vickers microhardness testing and shear strength testing were performed to estimate the mechanical properties of the Al/Mg bimetallic interfaces. The microhardness distribution of the Al/Mg bimetallic interfaces were measured perpendicular to the Al/Mg interface every 150 μm at a load of 300 g and a loading time of 15 s, from the A356 insert to the solidified AZ91D melt, with a Vickers hardness tester (200HV-5, Huayin, China). At present, no ASTM test standard exists for this kind of joint bimetal materials [38], so classical push-out testing was performed to investigate the shear strength of the Al/Mg bimetallic interface. The shear strength was tested with a material performance testing machine (AG-IC 100 kN, Shimadzu, Kyoto, Japan). The schematic diagram and principle for shear strength testing is shown in Figure 1c. The shear strength of the Al/Mg bimetallic interfaces is determined via Equation (2):

(2) $$S=\frac{F}{\pi dh}$$

where *S* is the shear strength of the Al/Mg bimetallic interfaces, *F* is the maximum force loaded during testing, *d* is the diameter of the A356 insert and *h* is the thickness of the tested specimen.

## 3. Results and Discussion

### 3.1. Macrostructure and Microstructure Evolution

#### 3.1.1. Macrostructure of the Al/Mg Interface

**Figure 2:**
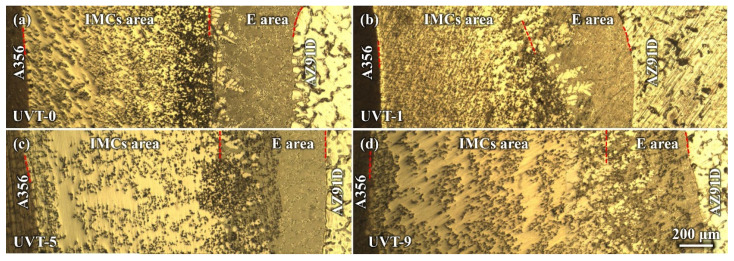
OM images showing the Al/Mg bimetallic interface of (**a**) UVT-0, (**b**) UVT-1, (**c**) UVT-5 and (**d**) UVT-9. IMCs: intermetallic compounds, E: eutectic. The red dashed lines illustrate the boundaries among A356, IMCs area, E area and AZ91D.

**Figure 3:**
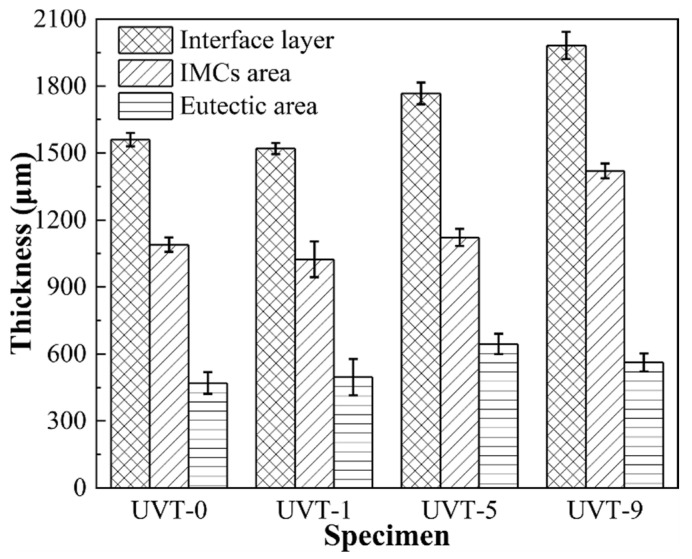
The respective thicknesses of the Al/Mg bimetal interface layer, IMC area and eutectic area. IMCs: intermetallic compounds.

The macrostructures of the Al/Mg bimetal were observed via OM, as shown in Figure 2a–d. It can be seen that an interface layer formed between the A356 and AZ91D. The interface layer consisted of an intermetallic compound (IMC) area near the A356 alloy side and a eutectic (E) area near the AZ91D alloy side. The average thickness of the Al/Mg interface layer, including the IMC area and eutectic area, of UVT-0, UVT-1, UVT-5 and UVT-9 was measured. For the UVT-0 specimen, the average thickness of the interface layer was 1560 μm, as shown in Figure 2a and Figure 3. With UVT, a transition zone between the IMC and eutectic areas appeared. In this work, this transition zone was divided into the E area. The average thickness of the interface layer of UVT-1 was almost unchanged, with a value of 1520 μm, as shown in Figure 2b and Figure 3. However, the average thickness of the interface layer showed an increasing trend as the UVT duration increased from 1 s to 9 s, as shown in Figure 2b–d and Figure 3. The average interface thickness of UVT-5 and UVT-9 was 1767 μm and 1982 μm, respectively, which increased by 13.3% and 27.1% compared with UVT-0, respectively.

The average thickness of the IMC area of UVT-0 and UVT-1 was 1090 μm and 1024 μm, respectively. As the duration of UVT increased from 1 s to 9 s, the thickness of the IMC area showed an increasing trend, as shown in Figure 3. The average thickness of IMC area of UVT-5 and UVT-9 was 1122 μm and 1420 μm, respectively. However, the thickness of IMC area was slightly changed in the UVT-0, UVT-1 and UVT-5 specimens and reached maximum in the UVT-9 specimen. The average thickness of the E area was not obviously changed in the UVT-0, UVT-1 and UVT-9 specimen and reached maximum for the UVT-5 specimen, with an average value of 645 μm. This indicates that UVT has an influence on the formation of both IMC and eutectic layers.

#### 3.1.2. Microstructure of the Al/Mg Interface

**Figure 4:**
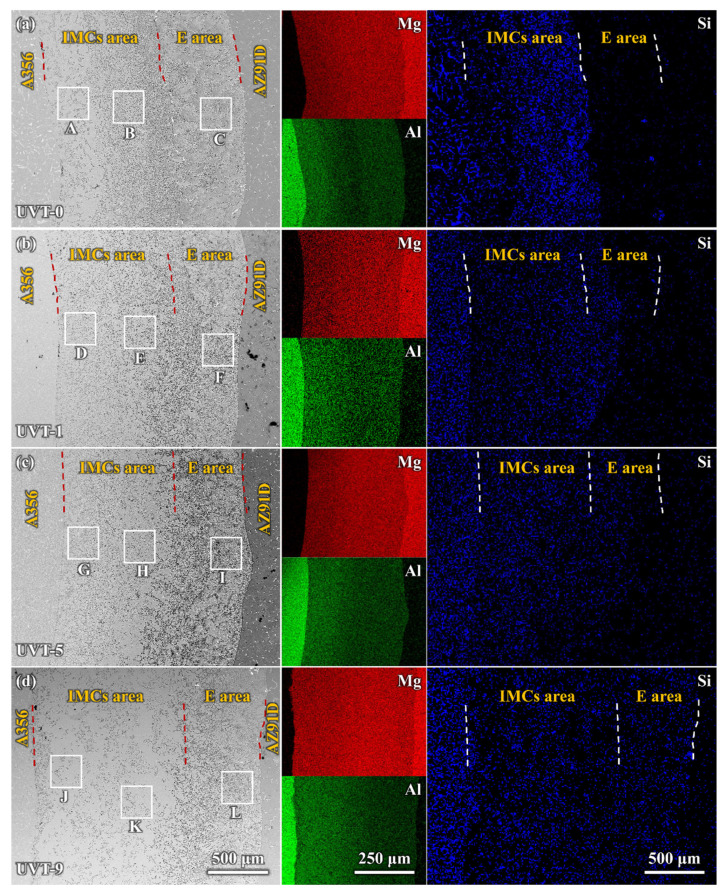
SEM images and EDS maps of the Al/Mg bimetal interface: (**a**) UVT-0, (**b**) UVT-1, (**c**) UVT-5 and (**d**) UVT-9. IMCs: intermetallic compounds, E: eutectic. The red and white dashed lines illustrate the boundaries among A356, IMCs area, E area and AZ91D. The uppercase letters A–L near the white boxes illustrate the regions.

To further study the details of phase distribution and the morphologies and sizes of the Al/Mg bimetallic interface after different UVT durations, SEM and EDS observations were conducted. As shown in Figure 4, a gradient distribution of Al and Mg elements was displayed. Mg was most abundant adjacent to the AZ91D side, while Al was most abundant adjacent to the A356 side, as shown in Figure 4a. The distribution of Si is interesting; Si was almost exclusively distributed at the IMC area and aggregated locally at interface of IMC area and E area for the UVT-0 specimen. Our previous studies also found similar results in as-cast Al/Mg bimetal fabricated via LFCC [19,35]. In the UVT-1, UVT-5 and UVT-9 specimens, the distribution of Si appeared at the E area and became uniform as the duration of UVT increased, as shown in Figure 4b–d. Additionally, the diffusion distance of Si increased with UVT. This indicates that UVT could decrease elemental segregation and accelerate the diffusion of elements, which would have a positive effect on the bonding properties of the Al/Mg interface.

**Figure 5:**
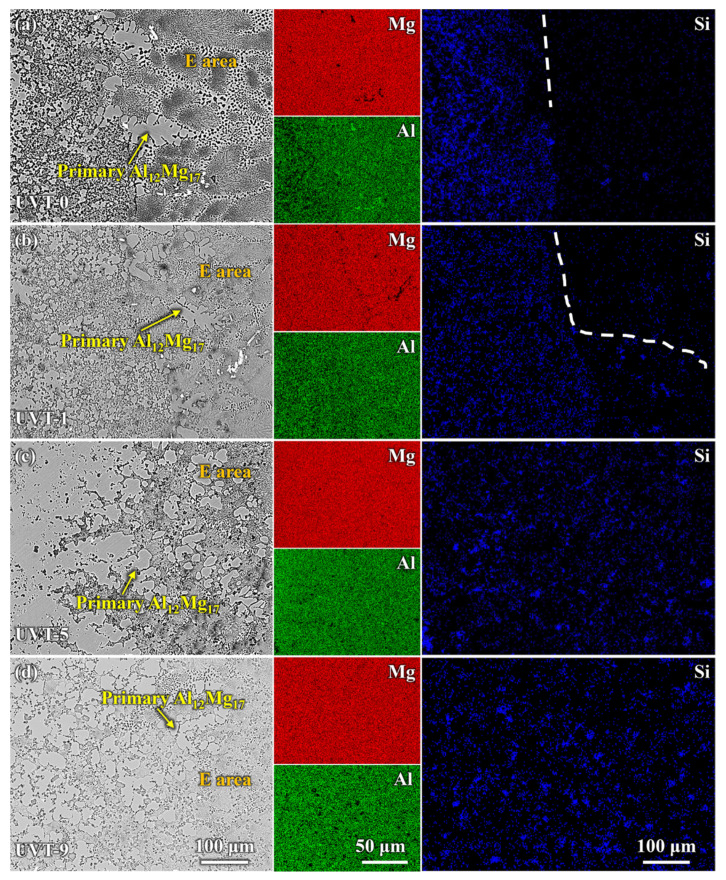
Enlarged SEM images and EDS maps of the transition zone between the IMCs and eutectic areas: (**a**) UVT-0, (**b**) UVT-1, (**c**) UVT-5 and (**d**) UVT-9. IMCs: intermetallic compounds. The white dashed lines illustrate the boundary of Si element at the transition zone.

The enlarged SEM images and EDS results of the transition zone between the IMC and eutectic areas are shown in Figure 5. A clear boundary can be seen between the IMC area and E area, and the distribution of Si was strongly restricted in the IMC area in the UVT-0 specimen, as shown in Figure 5a. Little Si was detected in the E area. A continuous Al_2_O_3_ film existed between the IMC area and E area, which hindered the diffusion of Si from the IMC to the eutectic. The oxide film was observed via TEM in our previous study [35]. As shown in Figure 5b, the distribution of Si partly expanded in the E area in the UVT-1 specimen, indicating that the continuous oxide film was partly removed by the UVT. When the UVT duration reached 5 s and 9 s, Si was not hindered by oxide film and was uniformly distributed in both the IMC area and E area near the transition zone (Figure 5c,d), indicating the oxide film was completely removed by UVT.

**Figure 6:**
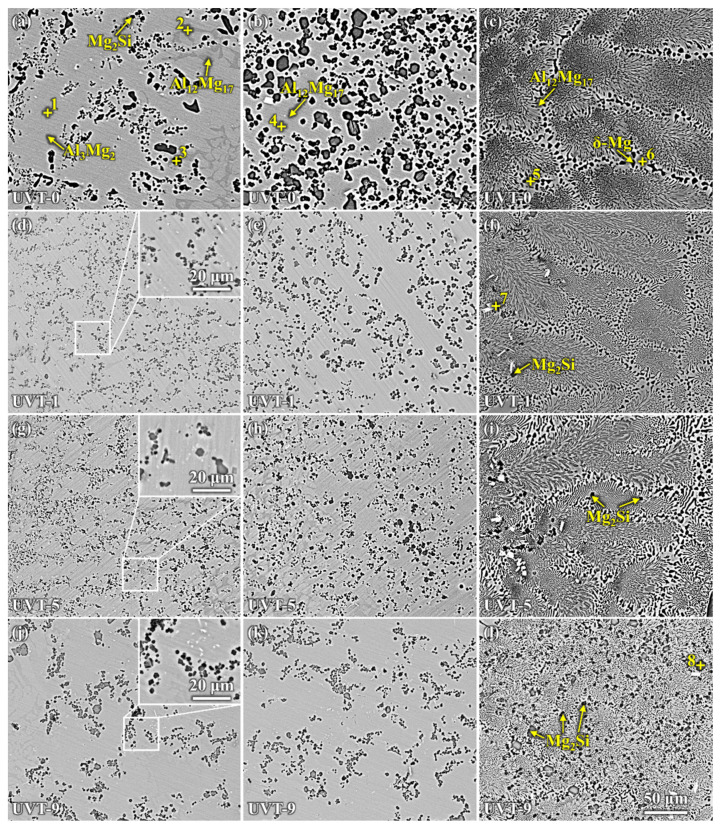
SEM images showing the microstructure of the Al/Mg bimetal interface: (**a**–**c**) corresponding to the marked region A–C in Figure 4 of UVT-0, (**d**–**f**) corresponding to the marked region D–F in Figure 4 of UVT-1, (**g**–**i**) corresponding to the marked region G–I in Figure 4 of UVT-5 and (**j**–**l**) corresponding to the marked region J–L in Figure 4 of UVT-9. The bigger white boxes in d, g and j are the enlarged images of the smaller white boxes.

Figure 6 shows the microstructure of the Al/Mg bimetal interface of the UVT-0, UVT-1, UVT-5 and UVT-9 specimens. According to the binary phase diagrams of Al-Mg [39] and Mg-Si [40], as well as the EDS point results shown in Figure 6 and Table 2, the interfaces of the Al/Mg bimetal were mainly composed of Al-Mg IMCs (β-Al_3_Mg_2_ and γ-Al_12_Mg_17_), δ-Mg + γ-Al_12_Mg_17_ eutectic and Mg_2_Si. As shown in Figure 6a–c and Table 2, it can be seen that the IMC area was mainly composed of IMCs (β-Al_3_Mg_2_ and γ-Al_12_Mg_17_) + Mg_2_Si, and the E area was composed primarily of γ-Al12Mg17 dendrites, δ-Mg + γ-Al_12_Mg_17_ eutectic structure and few Mg_2_Si particles in the UVT-0 specimen. Additionally, the IMC area could be divided into a β-Al_3_Mg_2_ +Mg_2_Si area and a γ-Al_12_Mg_17_ + Mg_2_Si area. However, the boundary between the two area is not clear. The β-Al_3_Mg_2_ +Mg_2_Si area is adjacent to the A356 insert, while the γ-Al_12_Mg_17_ + Mg_2_Si area is adjacent to the E area. The formation of β-Al_3_Mg_2_ and γ-Al_12_Mg_17_ is related to the Al and Mg atomic concentration. The EDS maps shown in Figure 4 indicate that Al was most abundant adjacent to the A356 side and showed a decreasing gradient, so that the β-Al_3_Mg_2_ rather than γ-Al_12_Mg_17_ was prone to form adjacent to the A356 insert. The Al atom concentration decreased, while the Mg atom concentration increased as the location grew more distant from the A356 insert, it is prone to form γ-Al_12_Mg_17_. The proportion of Mg_2_Si at the γ-Al_12_Mg_17_ area as 33.7%, higher than that at the β-Al_3_Mg_2_ area (11.7%), indicating that Mg_2_Si was segregated at the γ-Al_12_Mg_17_ area, corresponding to the OM image (dark particles in Figure 2a) and EDS map (Figure 4a). Most of the Mg_2_Si particles in the IMC area were worm-like and had an average size of 8.3 μm.

It can be seen that the Mg_2_Si particles at IMC area were refined in the UVT-1, UVT-5 and UVT-9 specimens, compared with the UVT-0 specimen, as shown in Figure 6d–l. The insert images in Figure 6d,g,j depict the Mg_2_Si particles, which are no more than 5 μm in size, polygon-shaped and homogeneously dispersed in the IMC area. The dispersed Mg_2_Si particles could deflect and bifurcate cracks during deformation, which would be beneficial for enhancing the bonding strength of the Al/Mg interface [35]. The polygon-shaped Mg_2_Si particles and the few short, rod-like Al_11_Mn_4_ phases were formed in the E area in the UVT-1, UVT-5 and UVT-9 specimens, as shown in Figure 6f,i,l and Table 2. The number of Mg_2_Si particles in the E area increased as the duration of UVT increased from 1 s to 9 s. In the UVT-9 specimen, the Mg_2_Si particles were homogeneously dispersed in the E area. It can be concluded that UVT could refine Mg_2_Si particles in the IMC area and promote the formation of Mg_2_Si in the E area.

**Figure 7:**
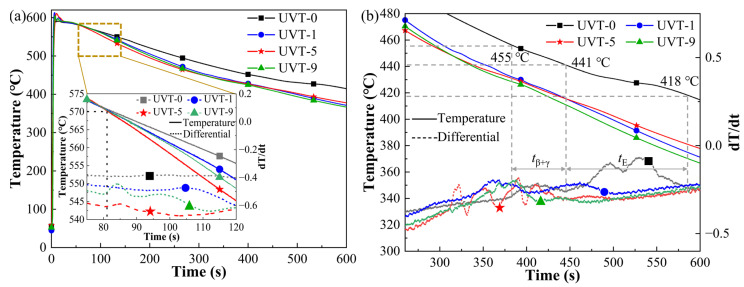
(**a**) Results of temperature measurement by thermocouple, (**b**) temperature curves of the interface formation during the cooling process.

To investigate and understand the formation of the Al/Mg bimetal interface, temperature measurements were conducted. Figure 7 shows the temperature curves and their differentials of the Mg/Al bimetal obtained via thermocouple on the surface of the A356 insert. As shown in Figure 7a, it can be seen that the cooling rate of UVT-1, UVT-5 and UVT-9 increased significantly after the ultrasonic device was activated. The cooling rate of UVT-0, UVT-1, UVT-5 and UVT-9 were calculated with values of 0.373 K/s, 0.447 K/s, 0.445 K/s and 0.457 K/s during the period of time between 83 s (570 °C) and 400 s, respectively. When the ultrasonic device was activated, the UVT system was in a resonant state. The A356 insert/radiator vibrated at a frequency of 20 kHz, and the acoustic cavitation and streaming flow in the melt near the ultrasonic radiator occurred [24,41]. As the UVT system was activated and continued, the cavitation bubbles and clouds nucleated, grew and collapsed. The formation and growth of cavitation bubbles absorbed and consumed heat from the melt [42], resulting in a higher cooling rate for UVT-1, UVT-5 and UVT-9, compared with UVT-0. Additionally, a gradient acoustic pressure was generated from the surface of the A356 insert to the AZ91D melt. The acoustic pressure near the surface of the A356 insert/radiator was at its maximum [41]. The acoustic streaming was formed by gradient acoustic pressure, which accelerated the heat transfer and increased the cooling rate of the AZ91D melt.

As shown in the insert image in Figure 7a, a rise in the differential curve at 83 s was observed in the UVT-5 and UVT-9 specimens. Haq et al. [43] indicated that a new phase forms as the differential increases, and the differential decreases when the phase transition ends. The cavitation bubbles collapse would create a local pressure of 100 MPa and a heating/cooling rate of >10^10^ °C/s [32], so that local supercooling occurred in the acoustic cavitation zone and promoted the nucleate of the AZ91D melt. This indicates that the nucleate of AZ91D melt leading to a rise in the differential curve at 83 s (570 °C) in the UVT-5 and UVT-9 specimens. It can also be seen that a UVT duration of 1 s is not enough for promoting the nucleate of AZ91D melt, as the differential curve at 83 s did not increase (insert image in Figure 7a).

Two main noticeable reactions occurred during solidification in UVT-0, as shown in Figure 7b. According to the Al-Mg binary phase diagram, it could be concluded that the Al-Mg IMCs (β-Al_3_Mg_2_ and γ-Al_12_Mg_17_) were formed at a temperature between 455 °C and 441 °C, during the interval of *t*_β+γ_ from 387 s to 445 s. The γ-Al_12_Mg_17_ + δ-Mg eutectic area was formed at a temperatures between 441 °C and 418 °C, during the interval of *t*_E_ from 445 s to 586 s. With the increase of UVT duration, the two main reactions during solidification did not change (i.e., the formation of Al-Mg IMCs and the formation of γ-Al_12_Mg_17_ + δ-Mg eutectic). This indicates that there was no new phase formed in the UVT-1, UVT-5 and UVT-9 specimens compared with the UVT-0 specimen, in accordance with the SEM and EDS results shown in Figure 4 and Figure 6. However, the formation temperatures and time of Al-Mg IMCs and eutectic in the UVT-0, UVT-1, UVT-5 and UVT-9 specimens are different, as they were affected by the cooling rate and solute concentration.

During the acoustic cavitation bubble collapse, a micro-jet of liquid melt rushed into the imploding bubbles at high speed (roughly 110 m/s) and created a local hotspot at high temperature (~5500 °C) and pressure (100 MPa) [32]. The collapse of the high-frequency cavitation bubbles also induced impetuous shockwaves in the acoustic cavitation zone, which could have fragmented the oxide film at the Al/Mg interface. Additionally, the direction of the acoustic streaming flow was ideal in removing the fragment oxide particles, since it was almost perpendicular to the insert/melt interface [24,41,44]. Therefore, the diffusion of elements was not inhibited by the oxide film and the diffusion rate of Al, Mg and Si were accelerated by the acoustic cavitation and streaming flow. It was found that a relatively short duration (1 s) of UVT was not effective in eliminating the oxide film completely (Figure 4b and Figure 5b). However, the Mg_2_Si particles in the IMC area were refined and dispersed by the acoustic cavitation. A small amount of Mg_2_Si particles were formed in the E area where the oxide film at the Al/Mg interface was removed from the UVT-1 specimen. The increase of UVT duration increased the circles of cavitation bubbles from nucleate to collapse. When the UVT duration reached 5 s, the diffusion of Si was not inhibited by oxide film, and Mg_2_Si particles were dispersed at E area (Figure 4c and Figure 5c), indicating the oxide film at the Al/Mg interface was removed completely. The thickness of the Al/Mg interface layer and the IMC area of UVT-5 was increased, indicating the surface of the A356 insert was eroded by the acoustic cavitation and shock waves of the AZ91D melt. When the UVT duration reaches 9 s, the oxide film at Al/Mg interface was completely removed. The diffusion distance of Si element increased, and Mg_2_Si particles was homogeneously dispersed at E area, as shown in Figure 4d, Figure 5d and Figure 6l. However, as the circles of acoustic cavitation and shock waves increase, the surface erosion of A356 insert for UVT-9 specimen was severer than UVT-5 specimen, the thickness of Al/Mg interface layer and IMC area was increased, as shown in Figure 2, Figure 3 and Figure 4. The formation of the Al/Mg interface layer, IMC area and E area with different thicknesses would cause a change in the temperature curves and its differential, as shown in Figure 7b.

### 3.2. Mechanical Properties

#### 3.2.1. Microhardness

**Figure 8:**
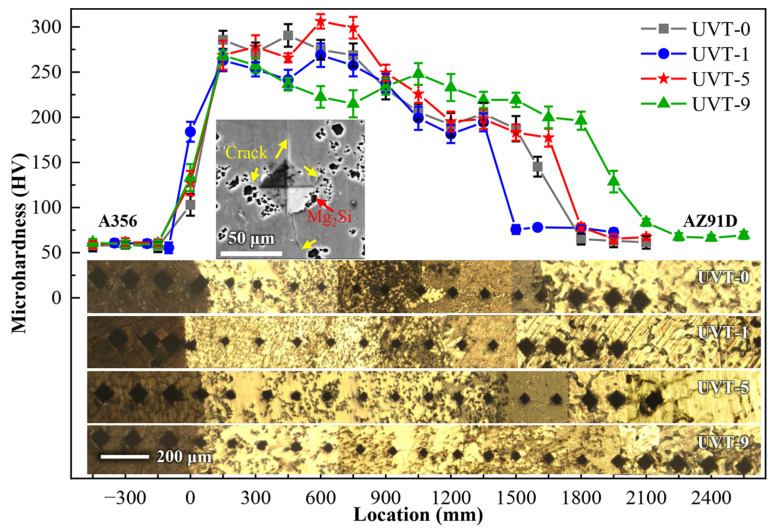
Vickers hardness of the Al/Mg bimetal interfaces. Insert OM images illustrate the location of the Vickers indentation. Insert SEM image illustrates the cracks and Mg_2_Si particles at the edges of Vickers indentation at IMC area.

The microhardness distribution at different locations of the Al/Mg interface layer is shown in Figure 8. It can be seen that the microhardness of A356 does not changed with UVT. The microhardness of the IMCs (220 HV–310 HV) exhibits a higher value than that of eutectic (187 HV–220 HV). The microhardness of AZ91D for UVT-1, UVT-5 and UVT-9 specimen is slightly higher than that for the UVT-0 specimen (65 HV), indicating that UVT has an effect on improving the microhardness of the AZ91D matrix. As shown in the insert SEM image in Figure 8, long cracks at edges of the Vickers indentation are visible, due to the IMCs with high microhardness being brittle. It is also noteworthy that the crack propagation was inhibited by the Mg_2_Si particles. The Mg_2_Si particles changed and inhibited the propagation path of cracks and increased energy consumption during crack propagation, which reduced the length of cracks. This indicates that the refined and homogeneously dispersed Mg_2_Si particles induced by UVT could effectively suppress the propagation of cracks during deformation, which is beneficial for improving the mechanical properties of Al/Mg bimetals.

#### 3.2.2. Shear Strength

**Figure 9:**
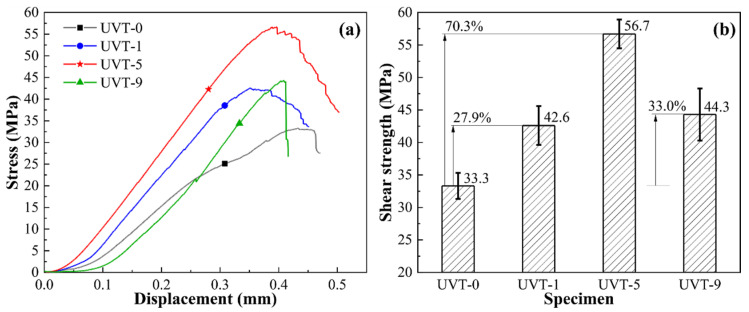
Results of shear strength testing: (**a**) stress-displacement curves and (**b**) shear strength of the Al/Mg bimetals.

Figure 9 shows the results of shear strength testing. The shear strength of the UVT-0 specimen is 33.3 MPa (Figure 9a,b), which is mostly consistent with our previous work [19]. As can be seen from the diagrams, the shear strength of UVT-1, UVT-5 and UVT-9 specimen were significantly enhanced. The shear strength of UVT-1, UVT-5 and UVT-9 specimen were 42.6 MPa, 56.7 MPa and 44.3 MPa, respectively. Compared with the UVT-0 specimen, the shear strength of UVT-1, UVT-5 and UVT-9 specimen were found to be enhanced by 27.9%, 70.3% and 33.0%, respectively. The shear strength of UVT-5 specimen was comparable to the Al/Mg bimetal enhanced with a Ni coating [15] and 19.4% higher than that of the Al/Mg bimetal processed via low-frequency vibration-assisted LFCC [19], indicating that the UVT is a cost-effective and high-efficiency technology for producing high-performance Al/Mg bimetallic components.

**Figure 10:**
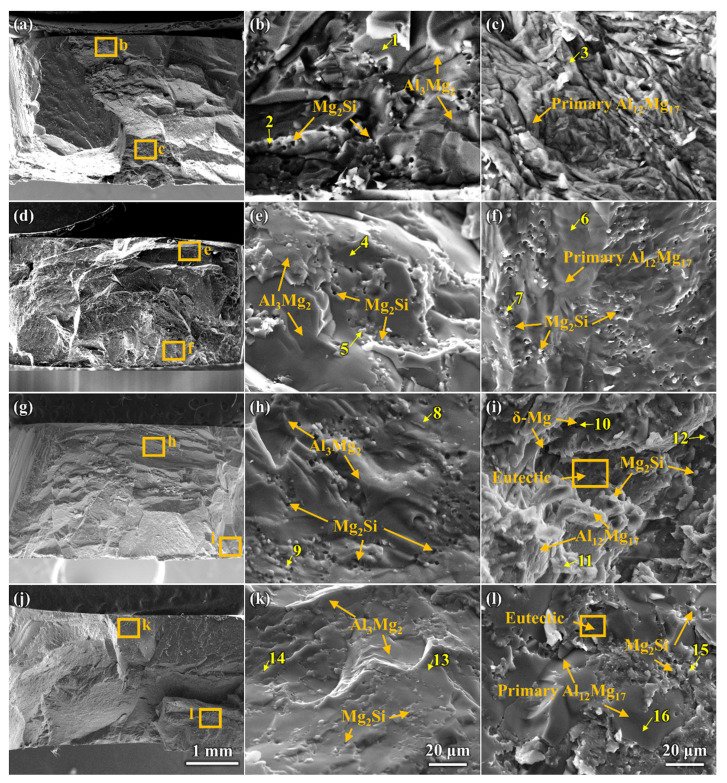
SEM images and EDS results showing the fracture morphologies and components analysis of the Al/Mg bimetals: (**a**–**c**) UVT-0, (**d**–**f**) UVT-1, (**g**–**i**) UVT-5 and (**j**–**l**) UVT-9. The numbers in the Figure indicate the locations of EDS point scans at the fractured surface.

To further investigate the fracture mechanism of Al/Mg bimetals with different UVT durations, fracture morphologies were observed and analyzed via SEM and EDS, as shown in Figure 10 and Table 3. As shown in Figure 10a,d,g,j, river patterns and cleavage planes could be observed on the fracture surfaces of UVT-0, UVT-1, UVT-5 and UVT-9, indicating that the fracture of Al/Mg bimetals are a type of brittle fracture. As shown in Figure 10b,e,h,k and Table 3, Al_3_Mg_2_ and Mg_2_Si were detected at the fracture surface of the Al/Mg bimetal interface, indicating the fracture occurred in the IMC area. The details of the fracture morphologies also showed river patterns and cleavage planes and indicated a typical brittle fracture in the IMC area. It is noteworthy that the Mg_2_Si particles had large sizes and gathered at the fracture surface of the IMC area in the UVT-0 specimen, while it was refined and dispersed in the IMC area of the UVT-1, UVT-5 and UVT-9 specimens, corresponding with the microstructure observation shown in Figure 6. As shown in Figure 10c and Table 3, river patterns of Al_12_Mg_17_ were detected, indicating that the UVT-0 specimen fractured only in the IMC area due to a brittle fracture form. As shown in Figure 10f and Table 3, river patterns and cleavage planes were observed at the fracture surface of the Al_12_Mg_17_ in the UVT-1 specimen, which also indicated a brittle fracture. However, dispersed Mg_2_Si particles at Al_12_Mg_17_ were observed. It can be deduced that the dispersed Mg_2_Si particles at IMC area and E area played a significant role in enhancing the shear strength of Al/Mg bimetal, as the Mg_2_Si particles could effectively suppress the propagation of cracks during deformation, which is depicted in Figure 8. However, the presence of oxide film between the IMC area and E area caused cracks to propagate along it [35]. As shown in Figure 10i and Table 3, Al_12_Mg_17_, Al_12_Mg_17_ +δ-Mg eutectic and Mg_2_Si were detected in the UVT-5 specimen, indicating the Al/Mg bimetallic interface fractures in the IMC area and E area. River patterns and cleavage planes are observed, but it shows a tendency to go from brittle fracture to ductile fracture, as the dimples of δ-Mg appeared. The shear strength of UVT-9 specimen decreased compared with the UVT-5 specimen, as shown in Figure 9. The thickness of the IMC area in UVT-9 increased by 26.6% compared with UVT-5, as cracks usually began and easily propagated in the brittle IMC area [9,45], which increased the length of the crack propagation path at IMC area, resulting in the decline of shear strength for the UVT-9 specimen. As shown in Figure 10l and Table 3, the Al_12_Mg_17_ + δ-Mg eutectic and Mg_2_Si were observed, indicating that the Al/Mg bimetallic interface of UVT-9 also fractured in the IMC area and E area. It could be concluded that the existence of oxide film and the increase of thickness for IMC area decreased the shear strength of the Al/Mg bimetal, while the removal of the oxide film, the refined and dispersed Mg_2_Si particles enhanced the shear strength of the Al/Mg bimetal.

**Figure 11:**
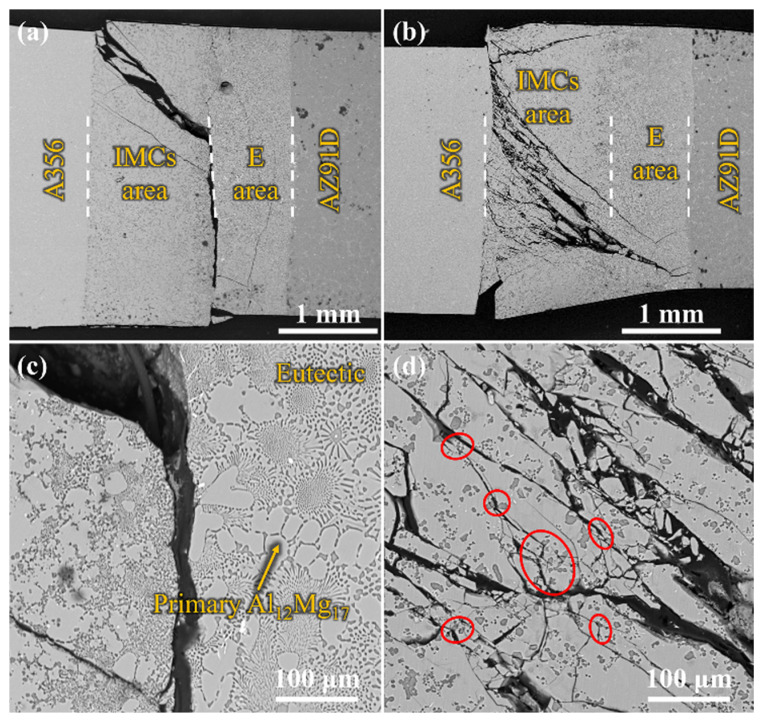
SEM images showing the crack propagation at the Al/Mg bimetallic interface after shear strength testing: (**a**,**c**) UVT-0, (**b**,**d**) UVT-5. The white dashed lines illustrate the boundaries among A356, IMCs area, E area and AZ91D. The red circles indicate the cracks are divided by the Mg_2_Si particles.

Figure 11 shows the crack propagation at the Al/Mg bimetallic interface after shear strength testing of UVT-0 and UVT-5. It can be seen that the cracks initiate at the IMC area, due to the IMCs are brittle and easy to crack during deformation [9]. It was found that the cracks propagated through the IMC area and expanded along the interface of the IMC area and E area in the UVT-0 specimen. The main cracks almost did not bifurcate to form micro-crack branches, so that the fracture morphology was relatively flat, which is depicted in Figure 10a–c and Figure 11a,c. The cracks passed through the IMC area and E area of the Al/Mg bimetal interface in the UVT-5 specimen, as shown in Figure 11b. This can be attributed to the removal of the oxide film, which improves the metallurgical bonding of the Al/Mg bimetallic interface [35]. Additionally, the refined and homogeneously dispersed Mg_2_Si particles could have hindered the propagation of cracks and divided the main cracks into several micro-cracks, as shown in Figure 8 and Figure 11d, which increased the energy consumption during crack propagation and improved the shear strength of the Al/Mg bimetal.

## 4. Conclusions

The effects of ultrasonic vibration treatment duration on microstructure and mechanical properties of the A356/AZ91D bimetallic interface processed via lost foam compound casting have been studied, and following conclusions are drawn:

1. The Al/Mg bimetallic interface consisted of IMC area (β-Al_3_Mg_2_ + γ-Al_12_Mg_17_ + Mg_2_Si) and E area (δ-Mg + γ-Al_12_Mg_17_ + Mg_2_Si); the application of UVT did not change the composition of phases at the interface. However, the distribution of the phases, especially Mg_2_Si particles, became more uniform. With a UVT duration of 1 s, the thickness of the Al/Mg interface layer was almost unchanged, compared with the UVT-0 specimen. However, the thickness of the Al/Mg interface layer and IMC area exhibited an increasing tendency with the increase of UVT duration.
2. Si mainly gathered in the IMC area of the UVT-0 specimen, but it could diffuse to the E area with UVT. Si and Mg_2_Si particles were more homogeneously dispersed in the IMC area and E area with the increase of UVT duration, which could be attributed to the removal of the oxide film. Additionally, the Mg_2_Si particles were refined by UVT via its acoustic cavitation and streaming flow effects, and the shape of them was changed to polygon-shaped from worm-like, with sizes of no more than 5 μm.
3. The IMCs exhibited the highest microhardness among the A356, AZ91D and eutectics. The microhardness of the Al/Mg bimetallic interface was not obviously changed with UVT. However, the cracks at the edges of the Vickers indentation were suppressed by the refined Mg_2_Si particles.

The shear strength of the Al/Mg bimetal increased and reached the maximum value of 56.7 MPa with the UVT duration of 5 s. It increased by 70.3% compared with the UVT-0 specimen (33.3 MPa). The Al/Mg bimetallic interface exhibited a typical brittle fracture morphology, while the fracture of UVT-5 specimen showed a tendency to go from brittle fracture to ductile fracture, as the dimples of δ-Mg appeared. This could be ascribed to the removal of oxide film and the refined and homogeneously dispersed Mg_2_Si particles, which improved the metallurgical bonding of the Al/Mg interface and suppressed the propagation of the cracks, respectively.

## Figures and Tables

**Table 1:** The chemical compositions (in wt.%) of the A356 and AZ91D alloys measured via energy dispersive spectroscopy (EDS).

	Mg	Al	Si	Ti	Mn	Fe	Zn
A356	0.96	Bal.	8.12	0.17	0.28	0.17	-
AZ91D	Bal.	9.08	-	-	0.41	-	1.08

**Table 2:** EDS results of different locations in Figure 6a–l.

Point No.	Element Compositions (at.%)				Possible Phase
	Mg	Al	Si	Mn	
1	41.23	58.77	-	-	Al_3_Mg_2_
2	53.96	46.04	-	-	Al_12_Mg_17_
3	59.39	-	40.61	-	Mg_2_Si
4	57.22	42.78	-	-	Al_12_Mg_17_
5	63.36	36.64	-	-	Al_12_Mg_17_
6	80.55	19.45	-	-	δ-Mg
7	5.35	72.23	-	22.42	Al_11_Mn_4_
8	65.63	-	34.37	-	Mg_2_Si

**Table 3:** EDS results at different locations on the fracture surface in Figure 10.

Point No.	Element Compositions (at.%)			Possible Phase
	Mg	Al	Si	
1	35.44	64.36	-	Al_3_Mg_2_
2	47.81	30.38	21.81	Mg_2_Si
3	60.06	39.94	-	Al_12_Mg_17_
4	42.29	57.71	-	Al_3_Mg_2_
5	48.24	40.03	11.73	Mg_2_Si
6	62.63	37.37	-	Al_12_Mg_17_
7	51.67	24.99	23.34	Mg_2_Si
8	41.47	58.53	-	Al_3_Mg_2_
9	40.11	51.80	8.09	Mg_2_Si
10	85.77	14.23	-	δ-Mg
11	61.54	38.46	-	Al_12_Mg_17_
12	47.65	24.96	27.38	Mg_2_Si
13	36.43	63.57	-	Al_3_Mg_2_
14	40.40	46.49	13.11	Mg_2_Si
15	56.90	10.61	32.49	Mg_2_Si
16	54.20	45.80	-	Al_12_Mg_17_

## Data Availability

Not applicable.
